# A Central Role for Ischemia and OCTA Metrics to Follow DR Progression

**DOI:** 10.3390/jcm10091821

**Published:** 2021-04-22

**Authors:** José Cunha-Vaz

**Affiliations:** 1 AIBILI—Association for Innovation and Biomedical Research on Light and Image, 3000-548 Coimbra, Portugal; cunhavaz@aibili.pt; Tel.: +351-239-480-136; 2 Coimbra Institute for Clinical and Biomedical Research (iCBR), Faculty of Medicine, University of Coimbra, 3000-548 Coimbra, Portugal

Diabetic retinopathy (DR) is a frequent complication of diabetes and through its vision-threatening complications, i.e., macular edema and proliferative retinopathy, may lead to blindness. DR is also the leading cause of vision loss in working age adults [1].

Recent advances in therapy, particularly intravitreal administration of anti-angiogenic agents, have opened new perspectives for vision recovery. However, the availability of treatment only for the late stages of the disease and its rate of success make it urgent to understand the early alterations of DR and their progression in order to develop timely treatments before vision loss.

When looking at the initial stages of DR it is said that it is present when microaneurysms and small hemorrhages appear on ophthalmoscopic examination. On histopathological examination, the first vascular changes occur in the small vessels in the form of endothelial proliferation, pericyte damage and microaneurysms [2]. Characteristically these initial lesions are focal and located at the posterior pole of the retina. With progression of disease the capillaries of the arterial side of the retinal circulation show increased vasoregression, with cell loss and closure, the number of microaneurysms increases and the areas of vessel closure enlarge. As they enlarge, they are seen to be crossed by remaining enlarged capillaries, which appear to act as arteriovenous shunts, receiving the blood directly from the surrounding closed vessel net [3]. Recent clinical studies using optical coherence tomography–angiography (OCTA) show that vessel closure occurs very early in diabetic retinal disease and is initiated in the macula [4]. Later on, as the disease progresses with remodeling of the retinal circulation and an altered retinal blood flow distribution probably through preferential arteriovenous preferential channels vessel closure develops and even appear to predominate in more peripheral regions of the retina [5].

Using the examination methods now available, it may be stated that the earliest alterations that may be detected clinically in the retina in diabetes are the breakdown of the blood–retinal barrier, vessel closure and alterations in the neurosensory retinal function. These alterations can be detected before ophthalmoscopic signs of DR are visible, in preclinical retinopathy [6].

Hyperglycemia appears to be sufficient to initiate the development of DR as revealed by the development of retinopathy in animals experimentally made hyperglycemic [7,8]. However, the observation that not all individuals with poor metabolic control develop advanced stages of DR suggests that other factors, such as local factors and genetic predispositions, are likely to determine individual susceptibility to the disease [9].

Diabetic retinopathy has been generally considered to be a microvascular complication of diabetes limiting the diagnostic and therapeutic focus to the vascular system. However, recent evidence has been accumulating suggesting that DR involves the neuronal as well as the vascular compartments.

The neurosensory retina has recently been shown to be altered very early in diabetes [10]. Together with reduced corneal nerve sensation and impaired autonomic innervation of the pupil, altered function of the retinal sensory nerve indicates that diabetes causes denervation of multiple sensory inputs to the eye. These are, therefore, strong arguments for a diabetic retinal neuropathy. However, it is now clear that these neuronal and microvascular changes occur in a different degree in different patients [6]. Furthermore, the link between neurodegeneration and microvascular disease remains open to question. Recent findings suggest that these two components of retinal disease due to diabetes may run independently [6].

It is recognized that the duration of diabetes and the level of metabolic control determine the development of DR. However, these risk factors do not explain the great variability that characterizes the evolution and rate of progression of the retinopathy in different diabetic individuals [9]. There are many diabetic patients who, after many years with diabetes, never develop vision-threatening retinal changes, whereas other patients progress rapidly. This is a message of major relevance when dealing with type 2 diabetes.

We have identified three major phenotypes of DR progression: one, characterized by slow progression, where neurodegeneration may be the only identified alteration and the retinal changes may be only a manifestation of the systemic neuropathy; a second one, characterized by occurrence of oedema resulting from breakdown of the blood retinal barrier which may occur, even in the absence of identifiable microvascular pathology; a third one identified by increased microaneurysm turnover and the presence of active microvascular lesions [10]. In a series of follow-up studies of 2 and 5 years, the first phenotype only rarely progressed to vision-threatening complications, whereas the second phenotype showed a relatively high risk for development of mild and manageable macular edema showed the higher risk for development of both clinically significant macular oedema and proliferative retinopathy. This phenotype appears to be also identified on OCTA by the presence of retinal vessel dropout [6]. Studies by our group confirm that capillary dropout and its extent appears to be the only retinal alteration that is directly correlated with the severity of DR [11].

It well accepted that only a subset of individuals with diabetes who develop retinal changes is expected to progress to advanced retinopathy stages and is at risk of losing functional vision during their lifetime.

A fundamental characteristic of DR is that its progression varies in different individuals with the development of vision-threatening complications occurring only in a few individuals. The activity of the disease and its progression vary from patient to patient making identification of biomarkers of progression of DR to vision-threatening complications a major need. Microaneurysm turnover calculated form fundus photography images and vessel closure measured by OCTA are major candidates to fulfil this role [12,13].

Ischemia, identified by retinal vessel closure, appears to be the central alteration occurring in DR and the one that determines its progression to more severe stages of the disease [11,14].

Ashton, who has contributed so extensively to our knowledge of DR, remarked in 1974 that ‘we must continue to look for more fundamental scientific investigations and at the same time develop new ways of examining the diabetic retina in an effort to unravel the still unsolved mysteries of diabetic retinopathy [15]. Metrics of retinal vessel density obtained with OCTA in a noninvasive manner, allowing repeated examinations, appear to identify the eyes/patients at a higher risk for increase in severity, thus promoting a larger role for precision medicine in the management of DR in individual patients [4,5,6,11].

Our group used a noninvasive, multimodal imaging approach to examine the prevalence of different disease pathways in the initial stages of the disease. Analysis of the variables representative of vessel closure, retinal neurodegeneration, and retinal edema showed a wide range of values in each ETDRS grade, demonstrating that there are very different degrees of vessel closure, neurodegenerative changes, and edema in eyes from different individuals with the same retinopathy grade.

When we examined the data for correlations between the parameters that represent vessel closure, retinal thinning, and edema, we found no association between these different alterations [6], confirming that different eyes in the same ETDRS severity level show different predominant disease pathways and that eyes from different individuals may have different phenotypes of disease progression.

Recently we have followed, in a longitudinal study, eyes categorized as minimal, mild, and moderate retinopathy using seven-field ETDRS grading. Of the three different disease pathways, only ischemia (vessel closure), identified by metrics of vessel density using OCTA, showed significant progression. Metrics of neurodegeneration and edema did not show progression over the period of follow-up, indicating that these two disease pathways do not appear to be associated with increased severity of the retinopathy.

One of the earliest changes associated with DR is, indeed, reduced vessel density, apparently due to a decrease in retinal blood flow in selected capillaries. The reduced capillary flow in the diabetic retina is most probably related to a decrease in the number of capillaries that carry red blood cells, instead of changes in capillary diameter, because skeletonized vessel density is the metric that better detects diabetic vessel closure. The increase in vessel closure due to the number of closed capillaries to red blood cell flow is compatible with the development of preferential channels or arteriovenous shunts which have been observed on histological and trypsin-digest preparations of diabetic retinas [3].

A study combining regional examination of the retinal circulation, including the macula region and the retinal midperiphery, in patients with type 2 diabetes in eyes with no retinopathy and different ETDRS grades of NPDR was performed using widefield Swept-Source OCTA (SS-OCTA). The study shows that retinal vessel closure is an early finding in the macular area of the diabetic retina and that this alteration increases as the retinopathy progresses in severity involving later more peripheral regions of the retina. Furthermore, SS-OCTA metrics of retinal vessel closure, allowing measurements to be performed in the macula and in more peripheral regions of the retina, may offer an objective and easier to perform alternative to ETDRS severity grading [5].

Regional distribution of the DR lesions in the retina may reflect risk factors and may be important in defining the stage of diabetic retinopathy [3,5,16]. Vessel closure in the midperiphery in a diabetic retina is indicative of an advanced stage of retinopathy, whereas vessel closure limited to the perifovea suggests a milder stage of the disease [5].

In conclusion, eyes in the initial stages of retinopathy in type 2 diabetic individuals show evidence of vessel closure and neurodegenerative changes, but these are present in different degrees in different individuals even when classified as belonging to the same ETDRS severity grade. Moreover, the metrics of these changes show a wide range of values. Definite neurodegeneration and vessel closure (i.e., ≥2 SD changes) can occur very early in the disease process but are not present in every patient and, when present, they do not have the same rate of progression and they appear to occur independently.

Furthermore, during a 3-year period of follow-up, one- or two-step worsening of retinopathy severity were associated with the presence of vessel closure and without neurodegeneration [17].

Vessel closure identified by OCTA shows different degrees of ischemia in individuals with the same ETDRS severity grade. This observation suggests that different patients with type 2 diabetes have different microvascular responses with some individuals being able to maintain a viable retinal circulation and showing minimal changes, whereas others respond with poor capillary recruitment and progressive vessel closure.

It is this variability of the retinal microvascular response in type 2 diabetes, regarding both their initiation and progression in relatively advanced stages of NPDR that we consider most relevant indicating that some individuals show steady and progressive worsening whereas others show a variable course and evidence of reversibility of their changes. These observations offer two important messages. First, the reversibility of the vessel closure opens the door for early intervention with the possibility of stopping disease progression. Second, each patient should be followed closely, and a variety of risk factors should be considered to determine a specific risk profile for that patient.

OCT-angiography metrics of retinal vessel closure, more specifically vessel density measurements based on skeletonized images, obtained in a noninvasive manner that allow repeated examinations and close follow-up, are a particularly promising candidate as a biomarker of DR severity progression and are expected to impact disease management.
